# Visual function improvement using photocromic and selective blue-violet light filtering spectacle lenses in patients affected by retinal diseases

**DOI:** 10.1186/s12886-017-0545-9

**Published:** 2017-08-22

**Authors:** L. Colombo, E. Melardi, P. Ferri, G. Montesano, S. Samir Attaalla, F. Patelli, S. De Cillà, G. Savaresi, L. Rossetti

**Affiliations:** 1 Department of Ophthalmology, San Paolo Hospital, University of Milan, Milan, Italy; 2 Department of Ophthalmology, AO Maggiore della Carità, Novara, Italy; 3 Associazione Retinitis Onlus, San Paolo Hospital, Milan, Italy

**Keywords:** Blue light, Retinitis pigmentosa, AMD, Glare, Led light, Contrast sensitivity

## Abstract

**Background:**

To evaluate functional visual parameters using photocromic and selective blue-violet light filtering spectacle lenses in patients affected by central or peripheral scotoma due to retinal diseases.

Sixty patients were enrolled in this study: 30 patients affected by central scotoma, group 1, and 30 affected by peripheral scotoma, group 2.

Black on White Best Corrected Visual Acuity (BW-BCVA), White on Black Best Corrected Visual Acuity (WB-BCVA), Mars Contrast Sensitivity (CS) and a Glare Test (GT) were performed to all patients.

Test results with blue-violet filter, a short-pass yellow filter and with no filters were compared.

**Results:**

All scores from test results increased significantly with blue-violet filters for all patients.

The mean BW-BCVA increased from 0.30 ± 0.20 to 0.36 ± 0.21 decimals in group 1 and from 0.44 ± 0.22 to 0.51 ± 0.23 decimals in group 2 (Mean ± SD, *p* < 0.0001 in both cases).

The mean WB-BCVA increased from 0.31 ± 0.19 to 0.38 ± 0.23 decimals in group 1 and from 0.46 ± 0.20 to 0.56 ± 0.22 decimals in group 2 (Mean ± SD, *p* < 0.0001 in both cases).

The letter count for the CS test increased from 26.7 ± 7.9 to 30.06 ± 7.8 in group 1 (Mean ± SD, *p* = 0.0005) and from 31.5 ± 7.6 to 33.72 ± 7.3 in group 2 (Mean ± SD, *p* = 0.031).

GT was significantly reduced: the letter count increased from 20.93 ± 5.42 to 22.82 ± 4.93 in group 1 (Mean ± SD, *p* < 0.0001) and from 24.15 ± 5.5 to 25.97 ± 4.7 in group 2 (Mean ± SD, *p* < 0.0001).

Higher scores were recorded with the Blue filter compared to Yellow filter in all tests (*p* < 0.05).

No significant differences in any test results could be detected between the Yellow filter and the No filter condition.

**Conclusions:**

The use of a combination of photocromic lens with a selective blue-violet light filter showed functional benefit in all evaluated patients.

## Background

Possible harmful effects of blue light have been known for half a century and linked to photochemical damage to retinal tissue and RPE [1, 2].

More specifically, some studies showed that, while longer blue wavelengths of the visible spectrum (465–495) are essential for circadian rhythm and a normal visual function [3–5], blue-violet light could cause an oxygen-dependent retinal injury acting on specific chromophores (by-products of visual cycle) [6, 7].

The chromophore *A2E* (*N-retinylidene-N-retinylethanolamine*) is considered the most important target molecule. Under blue-violet light exposure conditions (400–440 nm), this chemical seems to induce the production of reactive oxygen species and to initiate the activity of cysteine-dependent proteases, ultimately leading to apoptotis and cell death [6, 8–14].

One of the main known sources of blue light is the sunlight (25–35% of the emitted spectrum), but an increasing contribution from indoor illumination has been recorded in the last 10 years. The widespread commercialization of high brightness light-emitting diodes (LEDs) have strongly changed indoor daily illumination [15]. In addition, white-light LED (the most common type of LED) have became the most important technology for smartphone screens [16], tablet backlighted displays and other commonly used devices.

A significant part of a LED-emission spectrum peaks at 450 nm, thus within the wavelength range of blue light.

Shorter wavelength blue light has been reported to show a pivotal role in glare disability due to direct or reflected bright light and many attempts have been made to face this concern with intraocular or spectacle blue-light-filtering lenses.

Based on the results of Sahel and coworkers’ on the phototoxic action of blue light on human RPE cells [17], some optical brands have produced increasingly specific blue-light-filtering spectacle lenses.

We tested the commercially available *TRANSITION X-TRACTIVE®* plus *CRIZAL PREVENCIA®* (Essilor, France) spectacle lenses. The purpose of our study was to investigate whether people with ocular chronic disease characterized by either central or peripheral scotoma could benefit from their use, in terms of visual acuity, contrast sensitivity and glare.

## Methods

We recruited 60 consecutive patients from the San Paolo Hospital Eye Clinic in Milan, 30 with central scotoma (within the 5 central degrees from the fovea) and 30 with peripheral scotoma (outside the 5 central degrees from the fovea). Each patient underwent a full ophthalmologic examination. Specific functional tests were performed: Black on White Best Corrected Visual Acuity (BW-BCVA), White on Black Best Corrected Visual Acuity (WB-BCVA), Mars Contrast Sensitivity (CS) and a Glare Test (GT). BCVA scores were reported in decimals, while Contrast Sensitivity and Glare Test results were expressed as number of letters correctly identified by the patient. Except for the GT, all other tests were performed with standard charts (Snellen for BCVAs and Mars for CS). Al evaluations were performed monocularly. To avoid letter memorization we used different charts when testing with or without the filters, both for Snellen and Mars charts. The GT was performed using the testing procedure offered by the MonPack 3 device (Metrovision, Lille, FR): briefly, the test was performed at 1 m distance with a + 1.00 spherical added to the best correction for distance; a letter chart was displayed on the screen, with letters at increasing distance form a bright light source directed toward the patient; the number of letters recognized by the patient was then reported.

On each subject, we performed all the tests with and without the application of blue-violet light filtering lenses (*TRANSITION X-TRACTIVE®* plus *CRIZAL PREVENCIA®*, Essilor, France). Filter lenses come in 3 different colors (grey, brown and green) and, according to manufacturer instructions, the color selection was based on the individual subject preference in terms of visual comfort. These filtering lenses have two technologies: Transition Xtractive and Crizal Prevencia. The former is responsible for the photocromic effect. When activated, these photopigment dyes are able to filter 88–95% of light up to 430 nm. The latter is an antireflective coating providing protection against both UV and Blue-Violet light in the backside and frontside of the lens, filtering up to 20% of the blue-violet light.

All tests with blue-violet filters were performed after the activation of the lens by means of a UV light.

In order to compare the effect of the blue-violet filtering lenses with traditional short pass wavelength filters (yellow filter – 450 nm) a subgroup of 28 patients underwent the same tests with the two different filtering lenses and no filter lenses. Each patient was randomized to one of the 6 possible different testing sequences.

The current study was performed in compliance with the Declaration of Helsinki and international guidelines. Written informed consent was obtained from each participant.

All data were analyzed using linear mixed models that included nested random effects for subject and eye to account for correlation of repeated measures on the same eye (before and after the application of the filtering lenses) and from the same subject (left and right eye). CS and GT data were modeled as a count process with a Poisson error distribution.

## Results

We analyzed data from two sets of patients. The first set was composed of 30 patients with central scotoma: 19 with Age related Macular Degeneration (AMD), 3 with Stargardt disease, 2 with ocular albinism, 2 with Retinitis Pigmentosa (RP) and single cases of optic nerve atrophy, cone dystrophy, myopic degeneration and diabetic macular edema.

The second set was composed of patients with peripheral scotoma, 29 with RP and 1 with glaucoma. Mean age was 64 ± 21 for patients with central scotoma and 49 ± 13 for patients with peripheral scotoma. The demographics are summarized in Table 1.

**Table 1:** Demographic information: for each of the two groups (central or peripheral scotoma) the table reports the male to female ratio in the second column, the mean age in the third column and the distribution of the diseases causing the scotoma within each group

Demographics Scotoma	M:F	Age (Mean ± SD)	Disease	
Central (*N* = 30)	12:18	64 ± 21	*AMD* *Stargardt* *Cone Dystrophy* *Ocular Albinism* *Optic Nerve Atrophy* *Degenerative Myopia* *Retinitis Pigmentosa* *Diabetic CME*	19 3 1 2 1 1 2 1
Peripheral (*N* = 30)	13:17	49 ± 13	*Retinitis Pigmentosa* *Glaucoma*	29 1

Among patients, all blue-violet filter colors were uniformly represented and no significant association could be detected between the color and the type of scotoma (*p* = 0.82, chi-squared test). Linear mixed models were used to perform multivariate paired tests, with the inclusion of covariates (i.e. the filter color and the location of the scotoma), in order to compare test performances before and after the application of the filtering lenses.

Results are reported in Table 2. Both black on white and white on black visual acuity increased significantly following the application of blue-violet filter lenses. For patients with central scotoma the mean BCVA increased from 0.30 ± 0.20 to 0.36 ± 0.21 decimals for black on white letters and from 0.31 ± 0.19 to 0.38 ± 0.23 decimals for white on black (Mean ± SD, *p* < 0.0001 in both cases). For patients with peripheral scotoma the mean BCVA increased from 0.44 ± 0.22 to 0.51 ± 0.23 decimals for black on white letters and from 0.46 ± 0.20 to 0.56 ± 0.22 decimals for white on black (Mean ± SD, *p* < 0.0001 in both cases).

**Table 2:** Reports all test results before (column 3) and after (column 4) the application of filters

Test results, Mean (SD)		Without filters	With filters	Mean Subject Increase	*p*
Scotoma					
Central	BCVA (Black on White)	0.30 (0.20)	0.36 (0.21)	0.057 (0.03)	<0.0001
	BCVA (White on Black)	0.31 (0.19)	0.38 (0.23)	0.074 (0.045)	<0.0001
	Glare Test	20.93 (5.42)	22.82 (4.93)	4.52 (1.70)	<0.0001
	Contrast Sensitivity	26.7 (7.9)	30.06 (7.8)	3.37 (1.8)	0.0005
Peripheral	BCVA (Black on White)	0.44 (0.22)	0.51 (0.23)	0.074 (0.04)	<0.0001
	BCVA (White on Black)	0.46 (0.2)	0.56 (0.22)	0.098 (0.064)	<0.0001
	Glare Test	24.15 (5.5)	25.97 (4.7)	3.75 (1.3)	<0.0001
	Contrast Sensitivity	31.5 (7.6)	33.72 (7.3)	2.25 (1.62)	0.031

Column 5 reports the mean subject increase (expressed as the average of the individual means of the differences in the test results for the two eyes). All results are reported as Mean (SD). The last column reports the significance of each difference obtained from the linear models described in the Method section. Subjects are divided in patients with central scotoma (top white rows) and peripheral scotoma (bottom gray rows)

Significant increases were also detected in CS test: the letter count for the Mars test increased from 26.7 ± 7.9 to 30.06 ± 7.8 in patients with central scotoma (Mean ± SD, *p* = 0.0005) and from 31.5 ± 7.6 to 33.72 ± 7.3 in patients with peripheral scotoma (Mean ± SD, *p* = 0.031).

Glare was also significantly reduced, as shown by the results of the GT: the letter count for increased from 20.93 ± 5.42 to 22.82 ± 4.93 in patients with central scotoma (Mean ± SD, *p* < 0.0001) and from 24.15 ± 5.5 to 25.97 ± 4.7 in patients with peripheral scotoma (Mean ± SD, *p* < 0.0001).

Interactions between the location of the scotoma and the filter application were used to model the presence of a potentially differential change in patients with peripheral scotoma with respect to those with central scotoma, but in no case we could detect any significantly different behavior between the two groups in terms of performance increase.

Table 2 also reports the mean individual increase (expressed as the average of the subject means of the differences in the test results for the two eyes) in different test performances after the application of blue-violet filtering lenses. Of notice, all test results showed an increased performance in every subject tested except for a reduced white on black BCVA in a single patient with peripheral scotoma.

Table 3 reports the results of the comparison between the effect of blue-violet filter and a standard short pass yellow filter. A subgroup of 28 patients (15 with central scotoma and 13 with peripheral scotoma) was tested as previously described. Significant differences could be detected between the Blue-violet filter and the Yellow filter in all tests, with higher scores recorded with the Blue filter (*p* < 0.05). No significant differences in any test could be detected between the Yellow filter and the No filter condition.

**Table 3:** Reports the Estimated Difference and Standard Errors (SE) obtained by a randomized trial on NN patients

Test		Estimated Difference	SE	*p*
	Comparison			
BCVA (Black on White)	Blue-violet – Yellow	0.071	0.006	<.0001
	Blue-violet – No Filter	0.078	0.005	<.0001
	Yellow – No Filter	0.007	0.006	0.4197
BCVA (White on Black)	Blue-violet – Yellow	0.083	0.009	<.0001
	Blue-violet – No Filter	0.088	0.008	<.0001
	Yellow – No Filter	0.004	0.009	0.8778
Glare Test	Blue-violet – Yellow	0.186	0.041	<.0001
	Blue-violet – No Filter	0.204	0.041	<.0001
	Yellow – No Filter	0.018	0.043	0.9123
Contrast Sensitivity	Blue-violet – Yellow	0.089	0.036	0.0363
	Blue-violet – No Filter	0.103	0.035	0.0094
	Yellow – No Filter	0.014	0.037	0.9218

Each row block refers to a different test and each row within a block reports all possible differences in the test results between the Blue-violet filter, the Yellow filter and no filter correction. All *p*-values have been calculated with mixed linear models and corrected with the Tukey-Kramer method

## Discussion

The purpose of our study was to assess potential benefits derived from photocromic and selective blue-violet light filtering spectacle lenses in patients with central or peripheral scotoma.

### Short term visual function enhancement

Results from statistical analysis of our data show that patients wearing photocromic and selective blue-violet light filtering spectacle lenses could improve their visual performances.

In our study, we evaluated two distinct groups of patients: subjects with central scotoma, primarily affected by AMD, and subjects with peripheral scotoma, mostly due to a severe rod dysfunction in RP.

Despite the differences in the pathophysiological mechanisms of these retinal diseases and consequently in the damage localization within the retinal structures, we could find statistically significant improvements in both groups for each parameter: visual acuity (both *black on white* and *white on black*), contrast sensitivity and glare.

Glare is an important factor affecting the quality of life of patients affected by retinal dystrophies. In RP, for example, the RPE becomes inadequate to contrast light intraocular scattering. This, together with progressive death of rods and cones, leads to an increasing difficulty in facing light adaptation to light changes in the environment [18]. Photophobia and consequent glare disability are therefore unavoidable.

In dry AMD, on the other hand, affected patients experience a slow reduction in visual acuity due to macular photoreceptors death and RPE and choriocapillaris degeneration. This leads to the gradual appearance of a central scotoma in the visual field of involved eyes and glare becomes a significant impairment in everyday-life.

Blue-violet light is the interval of visible spectrum carrying the highest energy content and it has been considered the main responsible for glare vision since its discovery.

Technical specifications from *Essilor* about *Transitions XTRActive* lenses report a screening effect of the 88–95% for the outdoor blue-violet light and the 34% for the indoor radiation [19]. We can thus speculate that our observations may be a consequence of such a filtering property of blue light.

All patients tested, both with central or peripheral scotoma, did not complain about color vision distortion using the blue-violet filter, as opposed to yellow filters. Based the curve of sensitivity of the human eye, it might be hypothesized that such a selectivity for blue-violet light does not alter the colors perception.

Filtering lenses used in our study might have improved both visual acuity and contrast sensitivity by cutting a preponderant part of blue-violet glaring radiations, providing more favorable conditions for efficient visual signal coding from the residual photoreceptors.

To the best of our knowledge, no published studies have already explored this topic. Further investigations are needed to better understand the role of selective filtering lenses in improving quality of life in patients affected by different retinal diseases.

### Prevention in photoreceptor’s loss

AMD is the most important cause of visual impairment in the elderly population [20] and *dry-AMD* accounts for almost the 90% of the cases [21].

Even though increasing age, genetics and smoking are well established risk factors for AMD [22, 23], it is still not clear whether other aspects, including long-term exposure to short wavelength light, could contribute to the development of the disease [24–27].

This might have significant clinical implications, such as the preference for either blue-light or UVR only-filtering intra-ocular lenses in cataract surgery.

The dioptric system of the eye is able to filter UVR light, with the largest contributions from the cornea (for wavelengths below 300 nm) and the lens (300–400 nm or even larger spectrum intervals when age related opacity is present) [28, 29]. UVR can induce DNA breakdown and oxidative stress with the production of reactive oxygen species (ROS), which may cause oxidative damage to RPE cells and photoreceptors [30, 31].

Since the discovery of the *blue-light hazard,* blue light-filtering artificial lenses have been considered as a viable option for retinal protection, although the real impact of this procedure has not been definitely proven.

This might be an option for further protection in patients with retinal diseases undergoing cataract surgery at a young age, such as in RP patients, that would have to renounce the protection offered by their natural crystalline lens.

## Conclusions

Upon these considerations, a wider use of blue-violet light filtering spectacle lenses, especially in RP patients or old people with an increased risk of AMD progression, could not only represent an important instrument to face visual impairment due to retinal pathologies, but could play a complementary role in visual preservation.

Limits of our study are the small sample size and the short follow up time that does not allow observing the safety role of a lower retinal exposure to dangerous radiations by the years.

Well designed, long term studies could unveil which spectrum of visible light is to be filtered according with the human circadian rhythm and what is the real role of blue-violet light in the pathogenesis and the progression of many retinal disease, both degenerative and dystrophic.

## Abbreviations

AMD: Age related Macular Degeneration
BW-BCVA: Black on White Best Corrected Visual Acuity
CS: Contrast Sensitivity
GT: Glare Test
ROS: Reactive Oxygen Species
RP: Retinitis Pigmentosa
WB-BCVA: White on Black Best Corrected Visual Acuity
